# Natural Killer Cell Hypo-responsiveness in Chronic Lymphocytic Leukemia can be Circumvented In Vitro by Adequate Activating Signaling

**DOI:** 10.1097/HS9.0000000000000308

**Published:** 2019-10-30

**Authors:** Tom Hofland, Sanne Endstra, Calum K.P. Gomes, Renate de Boer, Iris de Weerdt, Vladimir Bobkov, Jurgen A. Riedl, Raimond Heukers, Martine J. Smit, Eric Eldering, Mark-David Levin, Arnon P. Kater, Sanne H. Tonino

**Affiliations:** 1 Amsterdam UMC, University of Amsterdam, Department of Experimental Immunology, Amsterdam Infection and Immunology Institute, Cancer Center Amsterdam, Meibergdreef 9, Amsterdam, the Netherlands; 2 Amsterdam UMC, University of Amsterdam, Department of Hematology, Amsterdam Infection and Immunology Institute, Cancer Center Amsterdam, Meibergdreef 9, Amsterdam, the Netherlands; 3 Amsterdam Institute for Molecules, Medicines and Systems (AIMMS), Division of Medicinal Chemistry, Faculty of Sciences, Vrije Universiteit, Amsterdam, the Netherlands; 4 Result Laboratory, Dordrecht, the Netherlands; 5 Department of Internal Medicine, Albert Schweitzer Hospital, Dordrecht, the Netherlands; 6 LYMMCARE, Lymphoma and Myeloma Center, Amsterdam, the Netherlands

## Abstract

Chronic lymphocytic leukemia (CLL) is characterized by an acquired immune dysfunction, which may underlie the hampered efficacy of cellular immunotherapy. Most data on dampened immune responses in CLL come from studies investigating CLL and T cell interactions. Natural killer (NK) cells may be an attractive alternative source of effector cells in immunotherapy in CLL, provided that functionality is retained within the CLL micro-environment. Despite their important role in anti-tumor responses, NK cells are not extensively characterized in CLL. Here, we studied the expression of activating and inhibitory receptors on CLL-derived and healthy control (HC) NK cells, and their functional response towards several stimuli.

NK cells from CLL patients have an increased maturation stage, with an expansion of NKG2C^+^ NK cells in CMV seropositive individuals. The cytotoxicity receptor NKG2D is downregulated, and the killing capacity through this receptor was markedly reduced in CLL-derived NK cells. In contrast, activation via CD16 (FCγRIII) led to adequate activation and functional responses in CLL-derived NK cells. These findings indicate that NK cells in CLL are not intrinsically defect and still perform effector functions upon adequate activating signaling. Clinical relevance of this finding was shown by treatment with novel nanobody-Fc constructs, which induced cytotoxic responses in both CLL- and HC-derived NK cells via CD16. Our results show that NK cells, in contrast to the T cell compartment, retain their function within the CLL micro-environment, provided that they receive an adequate activating signal. These findings warrant future studies on NK cell mediated immunotherapeutic strategies in CLL.

## Introduction

Chronic lymphocytic leukemia (CLL) is characterized by an acquired dysregulation of the immune system, which results in an increased risk of infections and decreased anti-tumor surveillance.^1,2^ Especially T cells have been shown to be dysfunctional in CLL, with reduced cytotoxicity, proliferative potential and impaired ability to form immune synapses.^3,4^ Several novel immunotherapies with impressive activity in lymphoid malignancies (such as immune checkpoint blockade, chimeric antigen receptor (CAR) transduced T cells, and bi-specific antibodies) show disappointing results in CLL.^5–9^ These disappointing responses might be caused by the reduced function of the effector T cells that are required for the therapeutic effect.^3,4^ It is therefore of interest to study other immune effector cells to determine their therapeutic potential and strategies to recruit them during immunotherapeutic strategies.

Natural killer (NK) cells play an important role in anti-viral and anti-tumor immune responses.^10^ NK cells do not express antigen-specific receptors, but instead are regulated by combined signaling through a variety of activating and inhibitory receptors.^11,12^ Despite their important role in antitumor immunity, little is known about NK cell phenotype or function in CLL. Data on the expression of several activating receptors such as NKp30, NKp46, DNAM-1, CD16 and killer-cell immunoglobulin-like receptors (KIR) on NK cells of CLL patients are inconsistent.^13–17^ One possible confounder that could explain inconsistent results on NK cell phenotype in CLL is cytomegalovirus (CMV) infection. CMV infection leaves a footprint on the phenotype of the NK cell compartment, leading to an increase in mature NK cells expressing the activating receptor NKG2C, which specifically recognize CMV infected cells, and expand after CMV reactivation.^18–24^ We have previously shown that CMV-specific CD4^+^ and CD8^+^ T cell subsets expand in CLL, whereas their anti-CMV activity is unaffected.^25–27^ The failure of other components of the immune system to control CMV may explain the expansion of CMV-specific T cells in CLL; for example reduced immunosurveillance by NK cells. However, it is currently unknown whether CMV-related NK cells are expanded in CLL patients, thereby further skewing the NK cell phenotype.

Similar to the phenotype of NK cells, there is discrepancy in data on the functionality of NK cells in CLL. Defects in NK cell cytotoxicity in CLL were first reported decades ago, although several papers since have also reported NK cell function to be unaffected in CLL.^13–17,28^ Discrepancies on NK cell function in CLL might be caused by the use of different experimental stimuli, *e.g.* via natural cytotoxicity receptors or antibody-dependent cellular cytotoxicity (ADCC) responses. If NK cell function in CLL is retained, NK cells could be exploited for cellular immunotherapeutic strategies such as bi-specific antibodies and chimeric antigen receptor (CAR) therapy.

Nanobodies (Nb) are single variable domains of heavy-chain only antibodies (VHH) derived from Camelidea (eg, camels and llamas). Nb have shown to be attractive therapeutic agents.^29,30^ By coupling Nb to human IgG1-Fc tails, CD16-mediated ADCC can be induced by these constructs.^31,32^ Recently a Nb-Fc construct has been described that targets the chemokine receptor CXCR4 (VUN401-Fc). VUN401-Fc has been shown to specifically target CXCR4, block interaction with the receptor and it is ligand CXCL12, and induce NK cell mediated ADCC.^31,32^ Current standard first-line therapy for CLL includes rituximab, a monoclonal antibody targeting CD20. However, CD20 is often only expressed at low levels on CLL cells, making it a suboptimal therapeutic target.^33^ Since CXCR4 is abundantly expressed by CLL cells,^34^ targeting this chemokine receptor may have more therapeutic potential.

To determine if autologous NK cells can be used for immunotherapy in CLL, we analyzed NK cell phenotype and function in CLL patients and age-matched healthy controls (HC). We found that NK cells are in an increased maturation state in CLL, which is further skewed by CMV infection. CLL-derived NK cells show reduced effector responses towards K562 cells, but are fully functional upon stimulation via CD16 in ADCC experiments. Anti-tumor responses by CLL-derived NK cells could also be induced by the CXCR4-targeting Nb-Fc construct VUN401-Fc, highlighting the therapeutic potential of these constructs. Our results indicate that CLL-derived NK cells, in contrast to the T cell compartment, retain intrinsic functionality within the CLL microenvironment. This warrants future research to incorporate NK cells into immunotherapeutic modalities to improve treatment responses in CLL.

## Results

### Expansion of CMV-responsive NK cells in CLL

**Figure 1:**
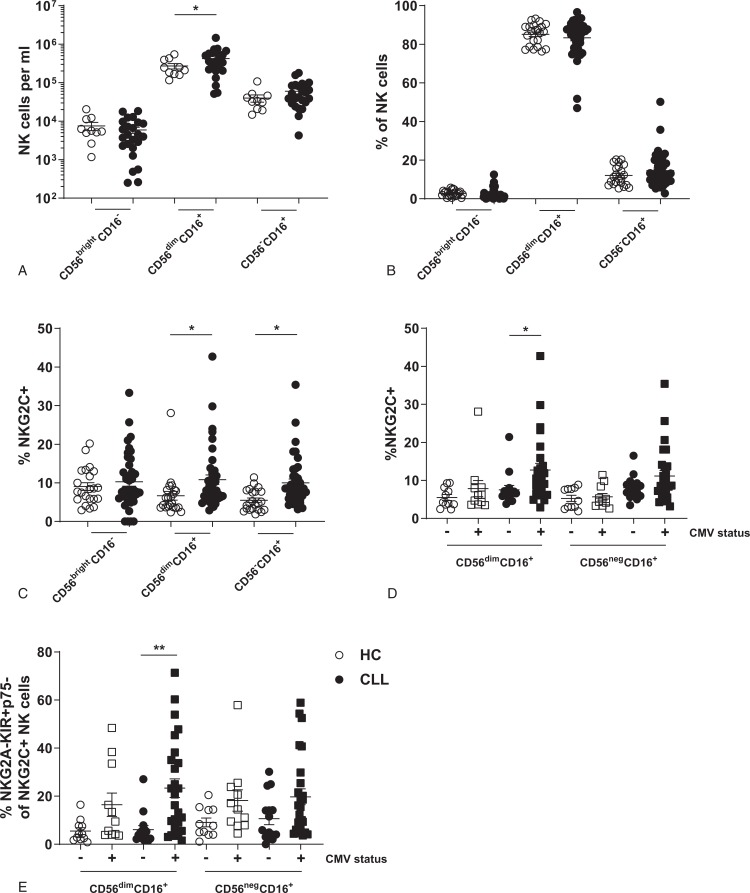
**Expansion of “CMV-specific” NKG2C**^**+**^** NK cells in CLL patients**. Subset composition of NK cells from HC (n = 22) or CLL (n = 41) was analyzed by flow cytometry. (A) Absolute number of NK cell subsets in peripheral blood of HC and CLL. (B) Relative subset distribution of NK cells. (C) Expression of NKG2C on NK cells of HC and CLL patients. (D) Expression of NKG2C on NK cells of HC and CLL patients stratified by CMV serology. (E) Frequency of NKG2C^+^ NK cells with a matured (NKG2A^-^KIR^+^p75^-^) phenotype. Bars indicate mean + SEM.^∗^ p < 0.05; ^∗∗^p < 0.01 (One-Way ANOVA).

NK cells can be divided into three subsets: CD56^bright^ cells that mainly produce cytokines upon stimulation, cytotoxic CD56^dim^ cells, and CD56^neg^ cells that have been reported to represent a dysfunctional NK cell subset.^11,35^ The CD56^dim^ subset dominates the blood compartment of NK cells, while numbers of CD56^bright^ and CD56^neg^ cells are low (Fig. 1A+B). Although CLL patients show a small increase in the absolute number of CD56^dim^ cytotoxic NK cells, the relative distribution of the different NK cell subsets does not change in CLL (Fig. 1A+B). To determine if CMV has an effect on the NK cell compartment in CLL, as has been shown for CMV-specific T cells,^25–27^ we measured the frequency of CMV-related NKG2C^+^ NK cells in CLL patients and HC. An increased proportion of CLL-derived NK cells express the activating receptor NKG2C (Fig. 1C). As expected, the expansion of NKG2C^+^ NK cells was confined to CLL patients that were CMV positive (Fig. 1D). NKG2C^+^ NK cells that expand after CMV infection have been shown to express low levels of NKG2A and p75, but high levels of KIR.^18,19,21,24^ Indeed, significantly more NKG2C^+^ NK cells in CMV positive CLL patients have this typical “CMV-specific” NKG2A^-^KIR^+^p75^-^NKG2C^+^ phenotype (Fig. 1E). Since these cells do not expand in CMV negative donors, we conclude that this expansion is driven by CMV instead of the malignant CLL cells. These data indicate an expansion of CMV-related NKG2C^+^ NK cells in CMV^+^ CLL patients.

### Subtle changes in expression of activating and inhibitory receptors on NK cells in CLL

**Figure 2:**
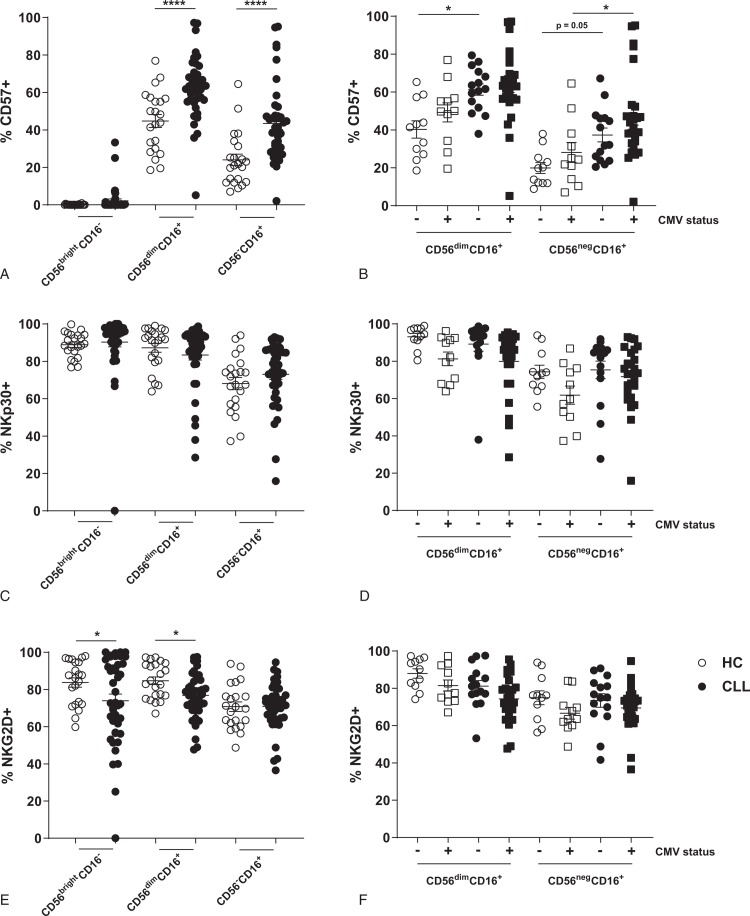
**Increased NK cell maturation but reduced expression of NKG2D on NK cells of CLL patients**. (A–C) Expression of CD57 (A+B), NKp30 (C+D) and NKG2D (E+F) on NK cells of HC and CLL patients, either in the global cohorts (A+C+E) or in both groups separated by CMV serology (B+D+F). Bars indicate mean + SEM.^∗^ p < 0.05; ^∗∗∗∗^p < 0.0001 (One-Way ANOVA).

In order to examine whether the expansion of CMV-responsive NK cells alters the NK repertoire in CLL, we studied the expression of other activating and inhibitory receptors on NK cells in CLL patients. CLL-derived NK cells in the CD56^dim^ and CD56^neg^ subsets display increased maturation in comparison to HC, as assessed by expression of CD57 (Fig. 2A). However, CMV status is not associated with NK cell maturation in CLL, indicating that CLL itself drives maturation of NK cells (Fig. 2B). Decreased expression levels of NKp30 on NK cells has been attributed to CMV infection.^20^ Reduced frequency of NKp30^+^ NK cells was related to CMV infection, but not to CLL in our cohort (Fig. 2C+D). Furthermore, CMV-responsive NKG2C^+^ NK cells in both CLL patients and HC show a NKp30^−^ phenotype in CMV seropositive donors, highlighting that reduced levels of NKp30^+^ NK cells in the global NK cell compartment of CLL patients can arise from the NK cell subset that associates with CMV infection (Supplemental Fig. 1A, Supplemental Digital Content). We also find CMV to influence the expression of CD160, p75 and ILT2 in HC and CLL (Supplemental Figure 1B-D, Supplemental Digital Content). Similar to others, we observe a reduced frequency of NK cells expressing the activating cytotoxicity receptor NKG2D in CLL patients (Fig. 2E).^13–17^ Although CMV infection seems to have a role in downregulation of NKG2D in HC and CLL, the frequency of NKG2D^+^ NK cells is also reduced in CLL patients not infected with CMV, suggesting a direct tumor effect on NKG2D expression (Fig. 2F). In contrast to the expression of these activating receptors, we find no significantly altered expression of the inhibitory receptors NKG2A, killer-cell immunoglobulin-like receptor (KIR) or KLRG1 on NK cells from CLL patients (see supplemental Fig. 2, Supplemental Digital Content). Thus, we find an increase of mature CD57^+^ CD56^dim^ NK cells in CLL patients, but the expression of most activating and inhibitory receptors on CLL-derived NK cells is similar to HC NK cells, except for NKG2D. Also, we demonstrate that the expansion of CMV-responsive NK cells can alter the expression of several activating and inhibitory receptors in CLL, highlighting the importance of determining CMV infection history when studying the NK cell phenotype.

### CLL-derived NK cells are hypo-responsive to K562 target cells, which is related to a lower expression of NKG2D

**Figure 3:**
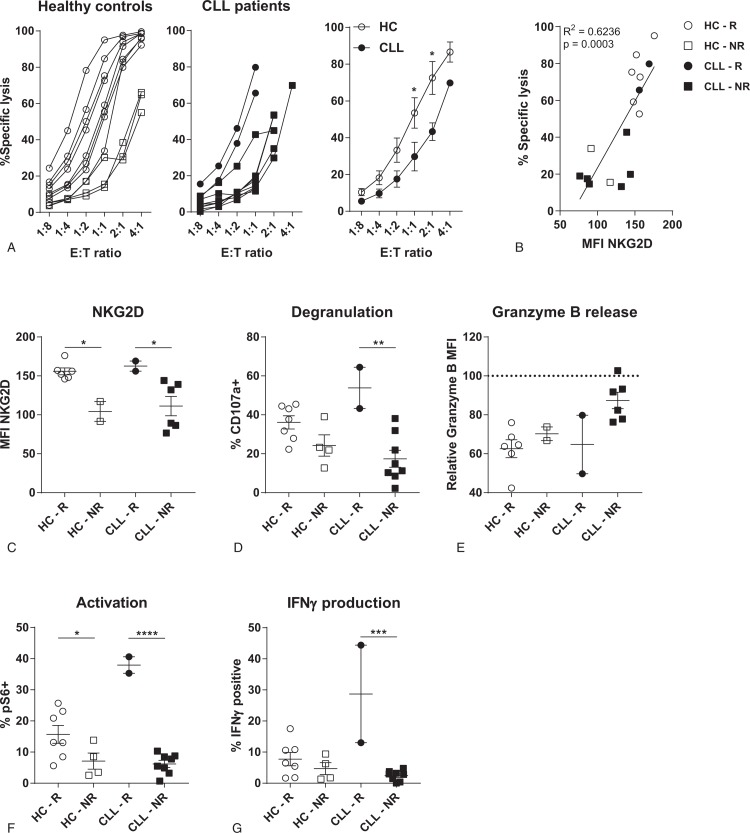
**CLL-derived NK cells are hypo-responsive**. HC- or CLL-derived NK cells were co-cultured with K562 target cells in indicated E:T ratio's (A), or in a ratio of 1:10 (B-G) for 3 to 4 hours. HC (n = 11) are indicated in white, CLL patients (n = 10) in black. In both groups good responders are depicted as circles, bad responders (<40–60% specific lysis in 1:1 or 2:1 E:T ratio) are depicted as squares. Target cell lysis and NK cell function were analyzed by flow cytometry. (A) Percentage target cell lysis for HC (left), CLL patients (middle) and groups combined (right). (B) Correlation between expression of NKG2D and percentage of target cell lysis at and E:T ratio of 1:1. (C) Expression of NKG2D on HC- or CLL-derived NK cells. (D) Percentage of degranulated (CD107a^+^) NK cells after co-culture with K562 target cells. (E) Release of granzyme B after co-culture with K562 cells. Granzyme B MFI was normalized to the MFI of NK cells that were not cultured with K562 target cells. (F) Activation of NK cells after co-culture with K562 cells, measured as the frequency of phospho-S6 high NK cells. (G) Percentage of IFNγ producing NK cells after co-culture with K562 cells. R = responder, NR = non-responder. Bars indicate mean + SEM.^∗^ p < 0.05; ^∗∗^p < 0.01; ^∗∗∗∗^p < 0.0001 (One-Way ANOVA and Mann-Whitney *U* test).

The increase in mature NK cells in CLL patients, especially with the cytotoxic NKG2C^+^ phenotype induced by CMV, is not in line with the reduced tumor surveillance in CLL patients. To determine functionality of NK cells in CLL patients, we studied NK cell responses towards K562 target cells, a classical NK cell target which activates NK cells through the lack of HLA expression. CLL-derived NK cells induced significantly lower target cell lysis compared to HC (Fig. 3A); 3 out of 11 HC donors had an inefficient cytotoxic response, while in CLL this was 8 out of 10 donors. Lack of cytotoxicity could not be explained by decreased expression of cytotoxic effector molecules, since expression levels of both granzyme B and perforin were equal (supplemental Fig. 3A+B, Supplemental Digital Content). As we found lower expression of the cytotoxicity receptor NKG2D on CLL-derived NK cells, and NKG2D signaling plays a role in the recognition of K562 cells,^36^ we correlated NKG2D expression with cytotoxicity. Expression of NKG2D correlated significantly with cytotoxicity towards K562, for both HC and CLL patients (Fig. 3B). NK cells of strong responders to the K562 cell line express higher levels of NKG2D, in both CLL as HC samples (Fig. 3C). Efficient cytotoxic responses correlated with higher levels of degranulation and a trend towards more release of granzyme B after contact with target cells (Fig. 3D+E). Activation of NK cells, measured as activation of the mTOR pathway by studying phosphorylation of S6, was lower in donors with low levels of NKG2D (Fig. 3F). mTOR signaling is essential to produce IFNγ.^37^ Indeed, we observed that production of IFNγ was decreased in donors with reduced phosphorylated-S6 levels (Fig. 3G). These results indicate that NK cell responses are reduced when expression of the activating receptor NKG2D is low, which is the case for the majority of CLL patients.

### Co-culture of HC-derived NK cells with CLL cells induces CLL-like dysfunction

**Figure 4:**
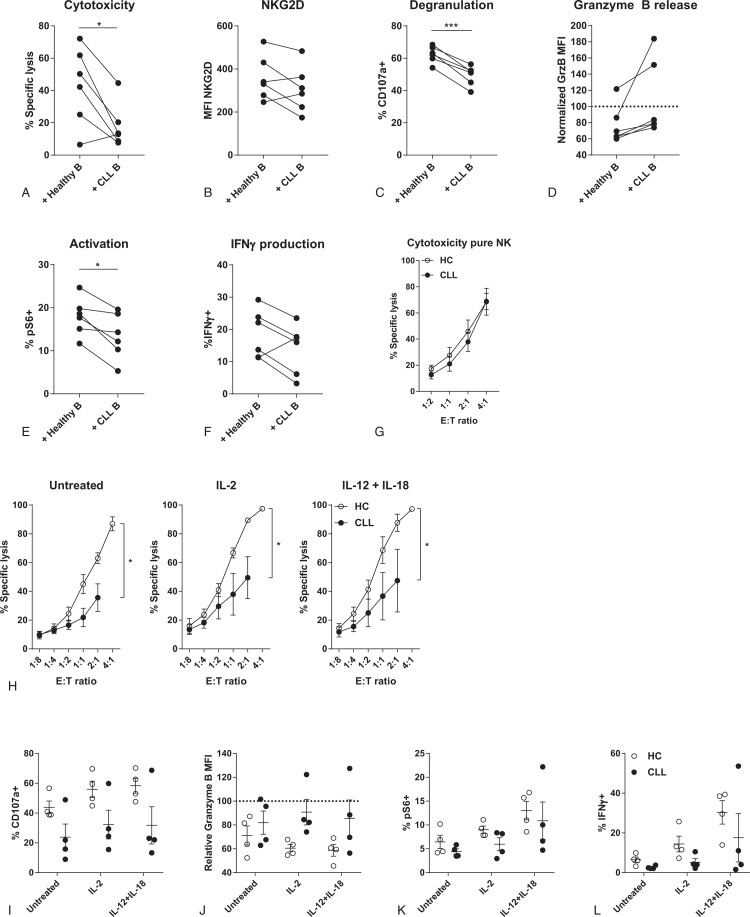
**CLL cells induce dysfunction in HC-derived NK cells, which is not reversible by pro-inflammatory cytokines**. HC NK cells were sorted and co-cultured with HC-B or CLL-B cells for 48 hours. Afterwards, K562 cells were added for 3 to 4 hours. Target cell lysis and NK cell function were analyzed by flow cytometry. (A) Cytotoxicity of NK cells after co-culture with either HC-B or CLL-B cells. (B) Expression of NKG2D on NK cells. (C) Percentage of degranulated (CD107a^+^) NK cells after co-culture with K562 target cells. (D) Release of granzyme B after co-culture with K562 cells. (E) Activation of NK cells after co-culture with K562 cells. (F) Percentage of IFNγ producing NK cells after co-culture with K562 cells. (G) CD56^+^CD3^-^ NK cells from HC and CLL patients were sorted and incubated with K562 target cells for 3 hours, and target cell death was analyzed by flow cytometry. (H–L) HC- or CLL-derived NK cells were stimulated overnight with IL-2 (100 U/ml), a combination of IL-12 (10 ng/ml) and IL-18 (100 ng/ml) or left untreated. Afterwards, cells were co-cultured with K562 target cells in indicated E:T ratio's (H) or in an E:T ratio of 1:10 (I-L) for 3 to 4 hours. (H) Specific lysis of K562 target cells induced by NK cells from HC and CLL patients. (I) Percentage of degranulated NK cells after co-culture with K562 target cells. (J) Release of granzyme B after co-culture with K562 cells. (K) Activation of NK cells after co-culture with K562 cells. (L) Percentage of IFNγ producing NK cells after co-culture with K562 cells. ^∗^p < 0.05; ^∗∗∗^p < 0.001 (Paired *t* test).

CLL tumor cells have been shown to directly influence the functionality of T cells.^3,38^ To assess whether CLL cells also directly impair the function of NK cells, we co-cultured sorted HC-derived NK cells with allogeneic healthy B-cells or CLL cells. After 48 hours of co-culture with CLL cells, NK cells induce significantly less target cell lysis compared to a co-culture with healthy B-cells (Fig. 4A). Decreased cytotoxic function of NK cells was accompanied by lower expression of NKG2D, reduced degranulation and less release of granzyme B (Fig. 4B–D). NK cells that were co-cultured with CLL cells also showed lower levels of activation and IFNγ production (Fig. 4E+F). These results indicate that CLL cells are able to directly hamper NK cell function towards K562 and create a hypo-responsive phenotype. The direct effect of CLL cells on NK cell function is further supported by the fact that pure fractions of sorted NK cells that are no longer in contact with CLL cells show similar cytotoxicity towards K562 cells as HC-derived NK cells (Fig. 4G).

### Stimulation with cytokines does not improve functionality of CLL-derived NK cells

We next studied whether the hypo-responsiveness of CLL-derived NK cells could be reversed by pro-inflammatory cytokines that boost NK cell functionality, like IL-2, IL-12, and IL-18. We stimulated HC and CLL PBMC fractions overnight with IL-2, or a combination of IL-12 and IL-18, before co-culture with K562 target cells. Pre-stimulation with activating cytokines did not lead to restoration of cytotoxicity of CLL-derived NK cells (Fig. 4H). Lack of cytotoxicity was reflected by reduced degranulation and release of granzyme B in the CLL-derived NK cells after co-culture with K562 cells (Fig. 4I+J). Stimulation with cytokines did not increase activation of NK cells in CLL, and IFNγ production after K562 stimulation was not restored (Fig. 4K+J). These results indicate that pro-inflammatory signaling is not enough to restore CLL-derived NK cell function.

### ADCC of CLL-derived NK cells is unaffected

**Figure 5:**
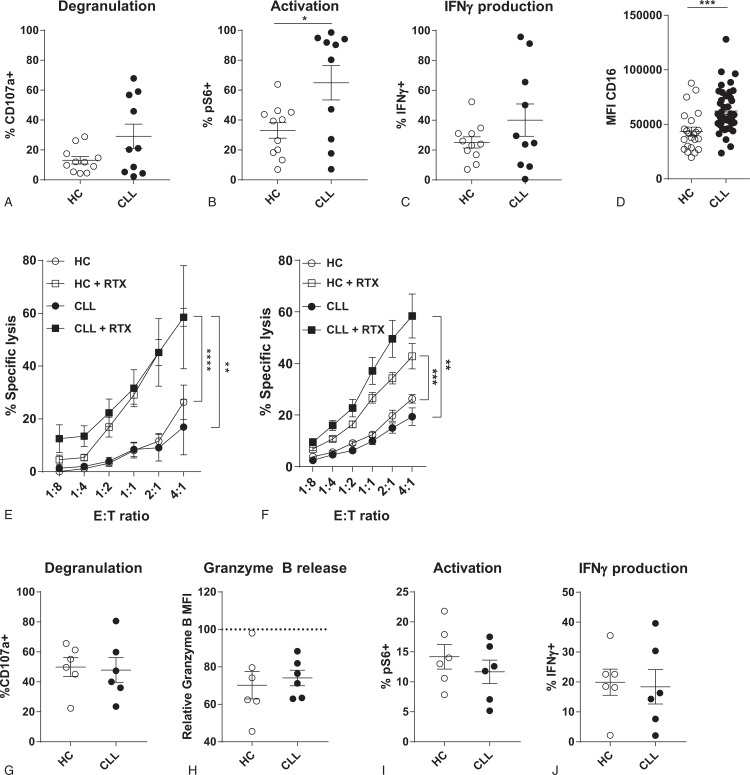
**CLL-derived NK cells are not intrinsically defective and can respond to opsonized target cells**. (A–C) HC- or CLL-derived NK cells were stimulated with PMA/Ionomycin for 4 hours. Afterwards, degranulation (A), activation (B) and IFNγ production (C) were measured by flow cytometry. (D-H) HC- or CLL-derived NK cells were co-cultured with untreated or rituximab pre-treated Daudi cells in indicated E:T ratio's (D), or in a ratio of 1:10 (E-H) for 3 to 4 hours. Daudi cells were opsonized by culturing with 10 μg/ml rituximab for 30 minutes at 37°C. Target cell lysis and NK cell function were analyzed by flow cytometry. (D) Expression of CD16 on HC- and CLL-derived NK cells, analyzed by flow cytometry. (E) Percentage specific target cell lysis induced by HC- (open symbols) or CLL-derived (filled symbols) NK cells towards Daudi cells. (F) Percentage specific target cell lysis induced by HC- (open symbols) or CLL-derived (filled symbols) NK cells towards MEC1 cells with and without rituximab opsonization. (G) Percentage of degranulated (CD107a^+^) NK cells after co-culture with rituximab treated Daudi cells. (H) Release of granzyme B of NK cells after co-culture with rituximab treated Daudi cells. (I) Activation of NK cells after co-culture with rituximab treated Daudi cells. (J) Percentage of IFNγ producing NK cells after co-culture with rituximab treated Daudi cells. Bars indicate mean + SEM. ^∗∗^p < 0.01, ^∗∗∗∗^p < 0.0001 (One-Way ANOVA and Paired *t* test).

Since the function of CLL-derived NK cells is hampered in settings where activation relies on natural cytotoxicity receptors (and especially NKG2D), we next studied whether this can be explained solely by the reduced expression of activating receptors, that is, NKG2D, or rather whether effector programs are intrinsically affected in CLL-derived NK cells. Stimulation of CLL-derived NK cells with a combination of PMA and Ionomycin, which mimics activation but bypasses any dependence on extracellular receptors, resulted in increased degranulation, activation and IFNγ production compared to HC NK cells (Fig. 5A–C), which demonstrates there is no intrinsic defect in effector programs in CLL-derived NK cells.

In contrast to the lower expression of NKG2D, we found that the Fc receptor CD16 was highly expressed on CLL-derived NK cells (Fig. 5D). We therefore hypothesized that recognition via CD16 can activate NK cells and trigger effector responses. To study the recognition of opsonized target cells, we co-cultured HC- or CLL-derived NK cells with Daudi and MEC1 cells that were pretreated with rituximab. Untreated Daudi and MEC1 cells are resistant to NK cell cytotoxicity (Fig. 5E+F). However, when target cells were pretreated with rituximab, NK cells are able to recognize and induce target cell lysis via ADCC. Both the HC- and CLL-derived NK cells are equally cytotoxic towards rituximab treated Daudi and MEC1 cells (Fig. 5E+F). Equal levels of cytotoxicity were reflected by a similar level of degranulation and release of granzyme B (Fig. 5G+H). Furthermore, NK cells of HC and CLL were equally activated after co-culture with rituximab treated Daudi cells, leading to a similar proportion of IFNγ producing NK cells (Fig. 5I+J).

These results further support that defects in NK cell functionality in CLL are not caused by intrinsic defects in effector pathways, and that adequate effector responses can be induced by receptors that are expressed in sufficient levels.

### CXCR4-targeting Fc-conjugated nanobodies induce similar ADCC responses by CLL- and HC-derived NK cells

Disappointing results with CD20-targeting antibodies can be explained by the low expression levels of CD20 on CLL cells.^33^ Other target molecules should be explored to increase therapeutic efficacy. The chemokine receptor CXCR4 is abundantly expressed on CLL cells.^34^ CXCR4 plays an important role in CLL pathobiology, as it mediates entry of CLL cells into lymph nodes, where they receive pro-survival stimuli.^39^ The high expression on the cell surface and the important role in CLL pathology make CXCR4 an attractive target for immunotherapy.

**Figure 6:**
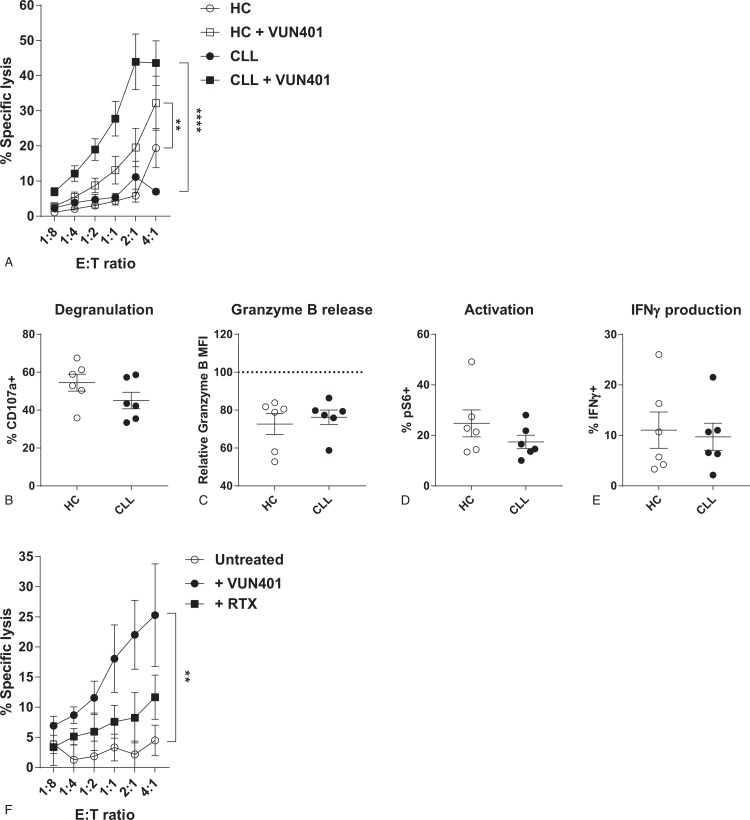
**Nanobody-Fc constructs are effective ADCC inducers in CLL and HC**. HC- or CLL-derived NK cells were co-cultured with untreated Daudi cells, or with Daudi cells pre-treated with VUN401, a CXCR4-Nb-Fc construct (1ng/ml) in indicated E:T ratio's (A) or in a E:T ratio of 1:10 (B–E) for 3 to 4 hours (n = 6). Target cell death, degranulation, granzyme B release, activation and cytokine production were measured by flow cytometry. (A) Percentage specific target cell lysis induced by HC- (open symbols) or CLL-derived (filled symbols) NK cells. (B) Percentage of degranulated (CD107a^+^) NK cells after co-culture with VUN401-treated Daudi cells. (C) Release of granzyme B of NK cells after co-culture with VUN401-treated Daudi cells. (D) Activation of NK cells after co-culture with VUN401-treated Daudi cells. (E) Percentage of IFNγ producing NK cells after co-culture with VUN401-treated Daudi cells. (F) Percentage specific lysis of CLL cells after VUN401 treatment and co-culture with autologous NK cells (n = 6). Bars indicate mean + SEM. ^∗∗^p < 0.01, ^∗∗∗∗^p < 0.0001 (One-Way ANOVA).

To study if we can induce ADCC responses in CLL-derived NK cells towards CXCR4, a recently described Nb-Fc construct (VUN401-Fc) that targets CXCR4 was used.^31,32^ We pre-treated Daudi cells with VUN401, and co-cultured them with HC- or CLL-derived NK cells. Similar to our ADCC experiments with rituximab, VUN401-Fc induced ADDC in both HC- and CLL-derived NK cells leading to target cell death (Fig. 6A). Degranulation and release of granzyme B was similar in HC- and CLL-derived NK cells after co-culture with VUN401-Fc opsonized Daudi cells (Fig. 6B+C). VUN401-Fc treated Daudi cells induced similar levels of NK cell activation and IFNγ production in CLL- and HC-derived NK cells. To determine the clinical potential of VUN401-Fc in CLL, we treated CLL cells with VUN401-Fc and co-cultured them with autologous NK cells. Treatment with VUN401-Fc induced lysis of autologous CLL cells via ADCC, more efficiently than rituximab (Fig. 6F), demonstrating that VUN401-Fc can induce autologous NK cell mediated anti-tumor responses.

## Discussion

We studied the phenotype and function of NK cells in CLL patients compared to HC. We find an expansion of CMV-related NKG2C^+^ NK cells in CMV-seropositive CLL patients, which skews the expression of activating and inhibitory receptors leaving a footprint on the global NK cell compartment. CLL-derived NK cells are hypo-responsive towards K562 target cells, which correlates with reduced expression of the activating receptor NKG2D, an effect that could be induced in HC-derived NK cells by co-culture with CLL cells. However, CLL-derived NK cells are fully functional in ADCC experiments with both rituximab and a novel Nb-Fc construct, demonstrating that intrinsic functionality of CLL-derived NK cells is unaffected within the tumor micro-environment. Furthermore, our results highlight the clinical potential of CXCR4-targeting nanobody constructs to direct NK cells to CLL cells, and demonstrate that autologous NK cells can be employed for immunotherapeutic strategies in CLL.

We find that the expansion of CMV-responsive NK cells can confound the expression pattern of activating and inhibitory receptors on the global NK cells compartment in CLL. CMV infection may therefore explain inconsistent reports on the expression of multiple receptors on NK cells in CLL, *e.g.* NKp30,^14,16,40^ and should be kept in mind when comparing NK cell phenotypes.

The increased magnitude of the CMV-related NKG2C^+^ NK cell subset is in line with what has been found for CMV-specific CD4^+^ and CD8^+^ T cells, which also expand in CLL.^25,26^ Although expansion of NKG2C^+^ NK cells and CMV-specific T cells could be explain by increased subclinical reactivations of CMV, this has not been elucidated yet. The decreased activation of NK cells by natural cytotoxicity receptors that we describe here may also impact the ability of NK cells to recognize and target CMV reactivations in CLL. Since NKG2D plays a major role in the recognition of CMV infected cells (highlighted by the many proteins CMV expresses to temper NKG2D ligand expression^41^), the downregulation of NKG2D on CLL-derived NK cells might have significant consequences for NK cell responses towards CMV in CLL. The expansion of functional CMV-specific T cells in CLL could be related to the impaired NK cell response towards CMV.^25–27^

We showed that CLL cells can directly influence the functionality of HC-derived NK cells. Several mechanisms could explain how CLL cells affect NK cell function. First, shedding of soluble NK cell ligands by CLL cells, as has been reported,^42,43^ can explain the reduced function of HC-derived NK cells after co-culture with CLL cells, although increased shedding of such ligands was not confirmed by all studies.^16^ A recent study proposed that production of TGF-β by CLL cells could be responsible for downregulation of NKG2D.^16,44^ Binding of activating ligands on the surface of CLL cells by direct cell contact can also play a role and lead to downregulation of activating receptors, which is not unlikely due to the high levels of tumor cells and NK cells in the peripheral blood. Future studies should shed more light on the contribution of both soluble and contact-dependent mediators of NK cell functionality in CLL.

Since several studies report only low levels of NK cell activation by CLL cells themselves, it seems that CLL tumor cells do not express enough ligands to trigger activating receptors on NK cells sufficiently to induce significant anti-tumor responses, or express enough evasive molecules to escape NK cell recognition.^14,16,42,43^ We show that the lack of NK cell responses to CLL is not related to an intrinsic functional defect, as we show unaffected ADCC by CLL-derived NK cells. We and others have shown NK-cell mediated ADCC towards autologous CLL tumor cells, and ADCC has been shown to be an important effector mechanism during the treatment with CD20 monoclonal antibodies.^14,16,45,46^ Since NK cells retain their functionality within the CLL micro-environment, in contrast to the T cell compartment, immunotherapeutic strategies in CLL should benefit from inducing NK cell responses with strong NK cell activators.

Since CD20 expression on CLL cells is often expressed at low levels,^33^ using other targets for ADCC that are universally expressed at high levels on CLL might be a better strategy. We tested a novel nanobody-Fc construct, VUN401-Fc, which targets the chemokine receptor CXCR4 and induces ADCC via the Fc receptor. CLL-derived NK cells show unaffected responses to VUN401-Fc and rituximab, but VUN401-Fc induced significantly more lysis of CLL cells compared to rituximab. Since Nb can also be used to block ligand binding sites on receptors or enzymatic active pockets, they have the potential to interfere with signaling processes important for tumor cells.^47^ For example, VUN401-Fc has been shown to block CXCL12 binding to CXCR4, leading to reduced chemotaxis via this receptor.^32^ Since CLL cells also use CXCR4 to migrate into lymph nodes, where they proliferate and receive anti-apoptotic stimuli, blocking CXCR4 migration could be beneficial to reduce entry of CLL cells into lymph nodes and sensitize CLL cells for cell death.^39^ By coupling the Nb to an Fc tail, VUN401 has a dual targeting function by taking CLL cells out of their tumor-supportive micro-environment while also sensitizing them for ADCC. Future studies should explore the therapeutic potential of these promising CXCR4-targeting constructs.

Since NK cells retain their functionality in CLL, other immunotherapeutic strategies might also incorporate autologous NK cells to attain better responses. So far, CAR T cell therapy has only shown limited results in CLL compared to other leukemia's.^5,9^ The immunomodulatory environment of CLL that hampers T cell functionality has been proposed to play a role in reduced responses to CAR T cell therapy.^5,39^ Since NK cells are able to retain functionality within this micro-environment, retargeting NK cells to tumor cells via CAR constructs might lead to significant beneficial responses in CLL. NK cells can also be directed towards tumor cells by bi- or tri-specific antibodies, called bi-(or tri-) specific killer engagers (BiKE, TriKE). Recently, several studies have reported the use of BiKE that bind CD16 on NK cells and simultaneously recognize tumor antigens. The tandem antibody AFM13, which recognizes CD16A and CD30, completed a phase 1 study in refractory Hodgkin lymphoma with promising results.^48^ With regard to CLL, a construct has been developed that targets CLL cells via CD19, but is coupled to the natural ligand for the NKG2D receptor ULBP2 instead of CD16, which also showed promising activity.^49^ Finally, a TriKE targeting CD16, CD19 and stimulating NK cells via IL-15 also showed promising activity in CLL-derived NK cells.^50^ Studies targeting NK cells with BiKE's and TriKE's in CLL should be elaborated further, and could include nanobodies for developing novel constructs.

In conclusion, we show that CMV leaves a footprint on the phenotype of NK cells in CLL. CLL-derived NK cells have unaffected intrinsic functionality, but require adequate activating signaling to perform effector functions. Since NK cells are not functionally affected by the CLL micro-environment, NK cells are a promising source of effector cells and future studies should aim to incorporate NK cells into novel immunotherapeutic strategies for CLL.

## Materials and methods

### Patient and healthy donor samples

Peripheral blood samples from untreated CLL patients were collected at the Albert Schweitzer Hospital in Dordrecht and the Amsterdam University Medical Centers, location AMC in Amsterdam. Age-matched healthy controls served as the control group: monoclonal B-cell lymphocytosis was excluded by CD19, CD5, κ, and λ immunophenotyping. HC were recruited at the Amsterdam University Medical Centers, location AMC in Amsterdam. In addition, buffycoats from healthy donors were obtained from Sanquin Blood Supply, Amsterdam. Patient characteristics, and details on the samples that were used in the various experiments, are presented in Table 1. Ethical approval was provided by the medical ethical committee at the Amsterdam University Medical Centers, location AMC in Amsterdam, and written informed consent was obtained in accordance with the Declaration of Helsinki.

**Table 1:**
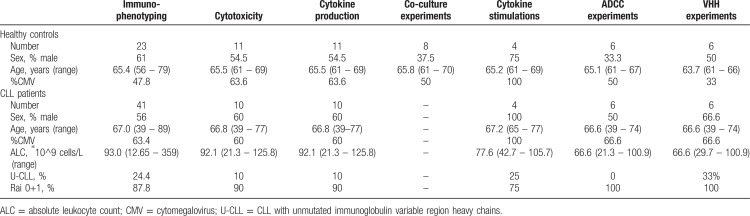
Patient Characteristics.

### Cell lines

K562 cells were cultured in Iscove's Modified Dulbecco's Medium (IMDM, ThermoFisher Scientific, Waltham, MA, USA), Daudi cells were cultured in RPMI 1640 medium (ThermoFisher Scientific). All medium was supplemented with 10% FCS and 1% penicillin/streptomycin.

### Phenotypic analysis of NK cells

Peripheral blood mononuclear cells (PBMC) from CLL patients and HC were isolated and cryopreserved as described earlier.^25^ To enrich for NK cells, samples from CLL patients were CD19-depleted using CD19 immunomagnetic microbeads (Miltenyi Biotec, Bergish Gladbach, Germany). Afterwards, PBMC were washed with ice-cold phosphate-buffered saline containing 0.5% bovine serum albumin (PBA) and stained for 30 minutes at 4°C with saturating amounts of CD56 APC-Alexa Fluor 750, CD16 ECD, CD158 (a,h) APC, CD158 (e1,e2) APC, CD158 (b1, b2, j) APC, p75 PE, NKG2A PE-Cy7, BTLA PC7, CD160 PE, ILT2 APC (Beckman Coulter, Brea, CA, USA), KLRG1 Alexa Fluor 488, NKG2D Pe-Cy7 (ThermoFisher Scientific), CD3 FITC, NKp30 APC, CD57 PerCP-Cy5.5 (Biolegend, San Diego, CA, USA), CD3 PE (BD Biosciences, Franklin Lakes, NJ, USA) and NKG2C Alexa Fluor 700 (R&D systems, Minneapolis, MN, USA). Cells were washed twice with PBA, resuspended, and analyzed on a BD FACSCanto flow cytometer. Data analysis was performed using Flowjo Mac Version 10. Gating strategy can be found in supplemental Figure 4 (Supplemental Digital Content).

### Cytotoxicity assays towards K562 cells

PBMC samples from both CLL patients and HC were enriched for NK cells by CD19 depletion; the percentage of NK cells was determined by flow cytometry, and cells were rested O/N in an incubator at 37°C. K562 cells were labeled with CellTrace Violet, according to the manufacturer's protocol (Invitrogen). The next day, PBMC were co-cultured with K562 cells in various NK:K562 ratio's in a 96-wells plate, spun down slowly, and incubated for 3 hours at 37°C. Afterwards, cells were stained with MitoTracker Orange (Invitrogen, Carlsbad, CA) for 25 minutes at 37°C, followed by staining with To-pro-3 (Invitrogen) for 15 minutes at room temperature. Cells were analyzed on a FACSCanto flow cytometer. Gating strategy can be found in supplemental Figure 5A (Supplemental Digital Content). Specific lysis of target cells was calculated as (% target cell death in co-culture sample - % cell death target cells alone)/(100 – % cell death target cells alone).

### Cytokine production by NK cells

PBMC samples from both CLL patients and HC were enriched for NK cells by CD19 depletion, the percentage of NK cells was determined by flow cytometry, and cells were rested O/N in an incubator at 37°C. The next day, cells were co-cultured with K562 or Daudi cells (NK:target ratio of 1:10) or stimulated with PMA/Ionomycin, or left untreated for 4 hours at 37°C in the presence of brefeldin A (10 μg/ml, Sigma, St Louis, MO), GolgiStop and CD107a PE-Cy7 (BD Biosciences). Afterwards, cells were washed with PBA, and stained with CD56 BUV395, CD3 V500 (BD Biosciences) and Live/Dead Fixable Red Stain (Invitrogen) for 30 minutes at 4°C. Then cells were washed with PBA, fixed and permeabilized (Cytofix/Cytoperm reagent, BD Biosciences) and stained intracellularly for 30 minutes at 4°C with IFNγ BV421, pS6 (Ser240/244, Cell Signaling, Danvers, MA) and granzyme B AF700 (BD Biosciences). Cell were washed, resuspended in PBA, and analyzed on a LSR Fortessa flow cytometer (BD Biosciences). Data analysis was performed using Flowjo Mac Version 10. Gating strategy can be found in supplemental Figure 5B (Supplemental Digital Content).

### Co-culture of NK cells with CLL or HC-derived B cells

NK cells of HC were sorted from PBMC using CD3 FITC and CD56 APC-Alexa Fluor 750 using a FACSAria cell sorter (BD Biosciences). NK cells were co-cultured with allogeneic CLL or HC-derived B cells (isolated by CD19 MACS) in a NK:B ratio of 1:10 for 48 hours. Then, cytokine and cytotoxicity assays with K562 cells were performed as described above.

### Cytokine stimulation of NK cells

PBMC samples from both CLL patients and HC were enriched for NK cells by CD19 depletion after which percentage of NK cells was determined by flow cytometry. Cells were stimulated overnight with IL-2 (100 U/ml, Peprotech, Rocky Hill, NJ, USA), or a combination of IL-12 (10 ug/ml, R&D) and IL-18 (100 ug/ml, R&D), or left untreated. The next day, cells were washed and cytokine and cytotoxicity assays with K562 cells were performed as described above.

### Rituximab or CXCR4-nanobody-Fc mediated ADCC

Rituximab was obtained from Roche (Woerden, the Netherlands). The development and production of the CXCR4-targeting nanobody-Fc construct was described previously.^31,32^ Daudi or CLL cells were labeled with CellTrace Violet according to manufacturer's protocol and pre-treated with rituximab (10 μg/ml) or CXCR4-nanobody-Fc (1 ng/ml) for 30 minutes at 37°C. Cells were washed, and co-cultured with PBMC fractions of CLL patients and HC in different NK:Daudi ratio's for 3 hours at 37°C. For cytotoxicity assays towards autologous CLL cells, CLL cells were isolated by CD19 MACS. Target cell death and cytokine production by NK cells was analyzed as described above.

### Statistics

Data were analyzed using Mann-Whitney *U* test or one-way ANOVA with Bonferroni correction as indicated. Statistical tests were performed using Graphpad Prism 6. Differences were considered statistically significant when p values were < 0.05. Data are presented as mean + SEM for scatter plots.

## Supplementary Material

Supplemental Digital Content
