# Supplementary material for: Natural Killer Cell Hypo-responsiveness in Chronic Lymphocytic Leukemia can be Circumvented In Vitro by Adequate Activating Signaling
Source: Hemasphere. 2019 Oct 30;3(6):e308. doi: 10.1097/HS9.0000000000000308 (PMC6924557; doi:10.1097/HS9.0000000000000308)
Supplement: Supplemental Digital Content [file hs9-3-e308-s001.pdf]

Suppl. Figure 1

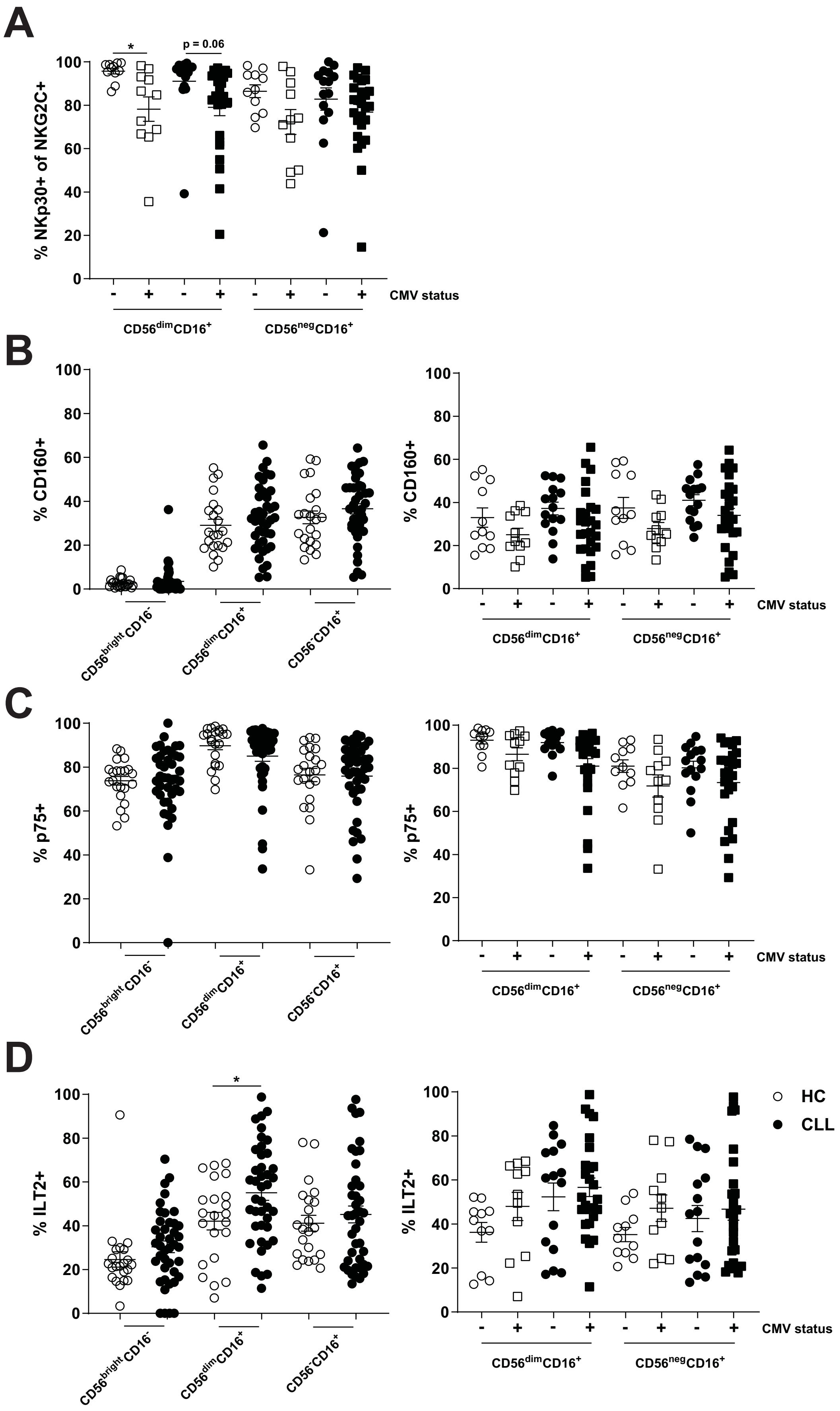

Supplemental Figure 1: Expression of surface receptors on NK cells in relation to CMV status

(A) Frequency of NKG2C^{+} NK cells expressing the activating receptor NKp30 (B-D) Expression of surface receptors CD160 (B), p75 (C) and ILT2 (D) on NK cell subsets from HC (n=22) or CLL (n=41) analyzed by flow cytometry. Left graphs show HC and CLL patients combined, right graphs show the groups split by CMV serology. Bars indicate mean + SEM. * = $p < 0.05$ (One-Way ANOVA).

Suppl. Figure 2

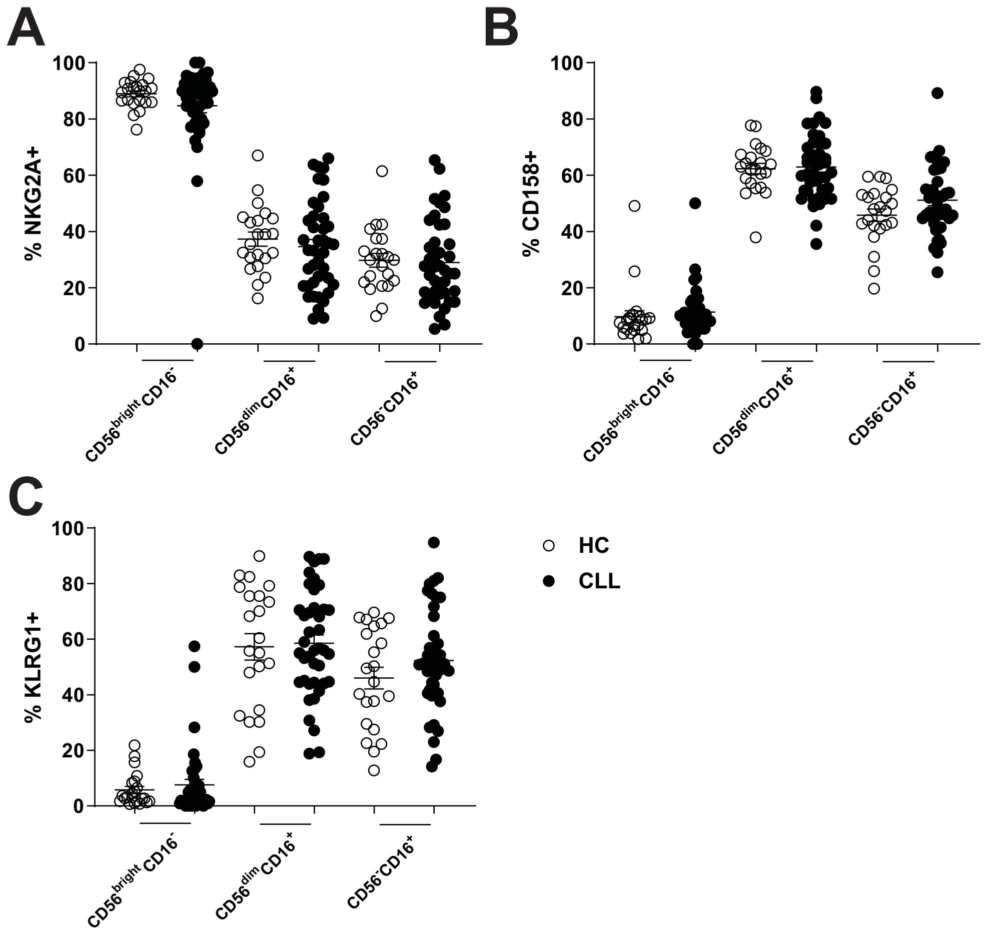

Supplemental Figure 2: Expression of inhibitory receptors on NK cells

Expression of inhibitory receptors NKG2A (A), KIR (B) or KLRG1 (C) on NK cell subsets from HC (n=22) or CLL (n=41) was analyzed by flow cytometry.

Suppl. Figure 3

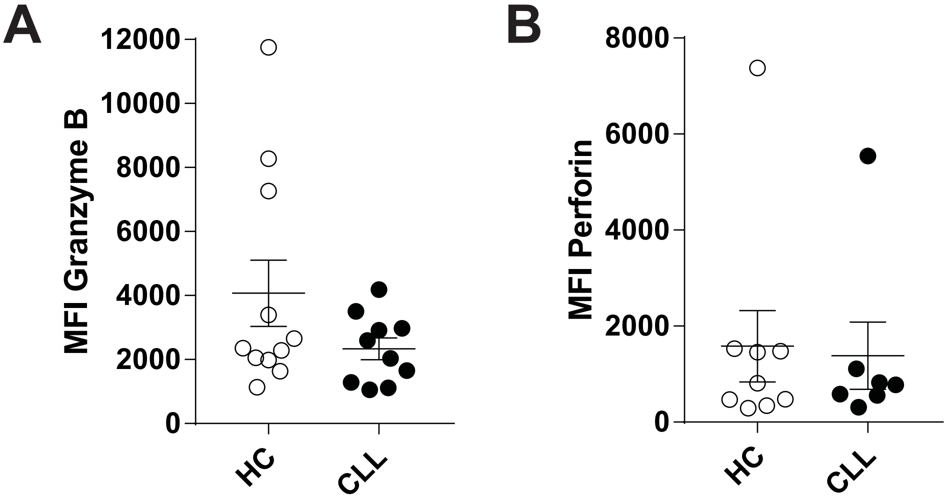

Supplemental Figure 3: Expression of cytotoxic molecules by NK cells from CLL and HC

Expression of cytotoxic molecules granzyme B (A) and perforin (B) by HC- or CLL-derived NK cells was analyzed by flow cytometry.

Suppl. Figure 4

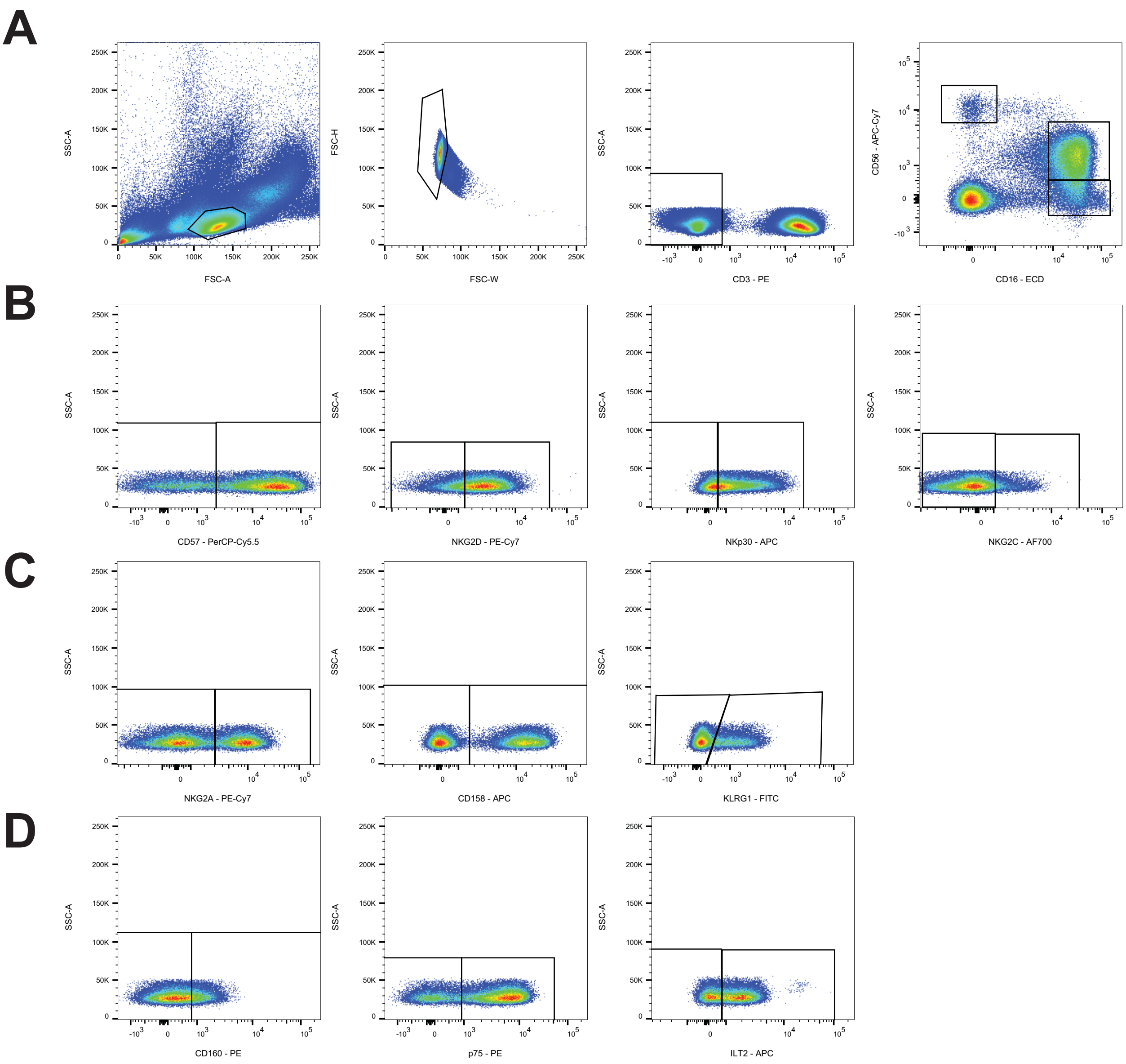

Supplemental Figure 4: Gating strategy for phenotyping of NK cells

Gating strategy for analysis of the expression of activating and inhibitory markers on NK cells from CLL patients and HC. (A) Gating of (from left to right) lymphocytes, single cells, $CD3^{-}$ negative cells, and NK cell subsets. (B) Examples of CD57, NKG2D, NKp30 and NKG2C staining and gating. (C) Examples of NKG2A, CD158 (combination of a,h,e1,e2,b1,b2 and j) and KLRG1 staining and gating. (D) Example of CD160, p75 and ILT2 staining and gating.

Suppl. Figure 5

A

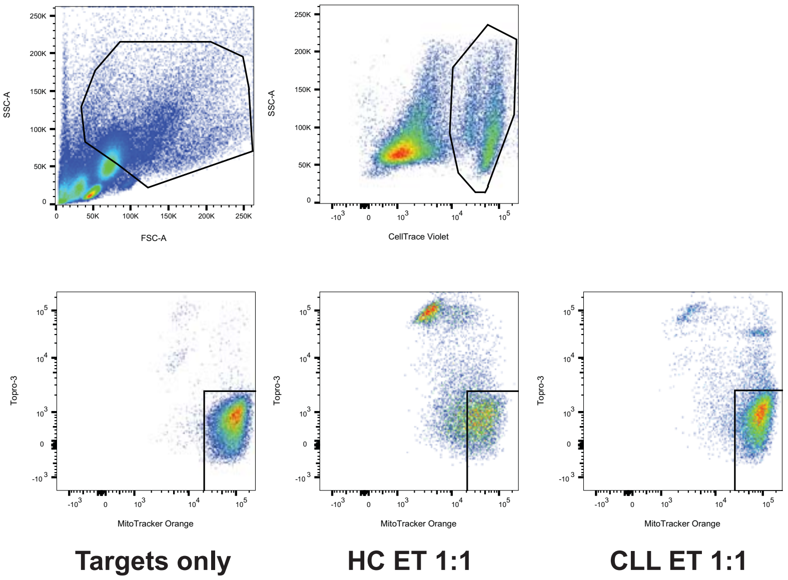

B

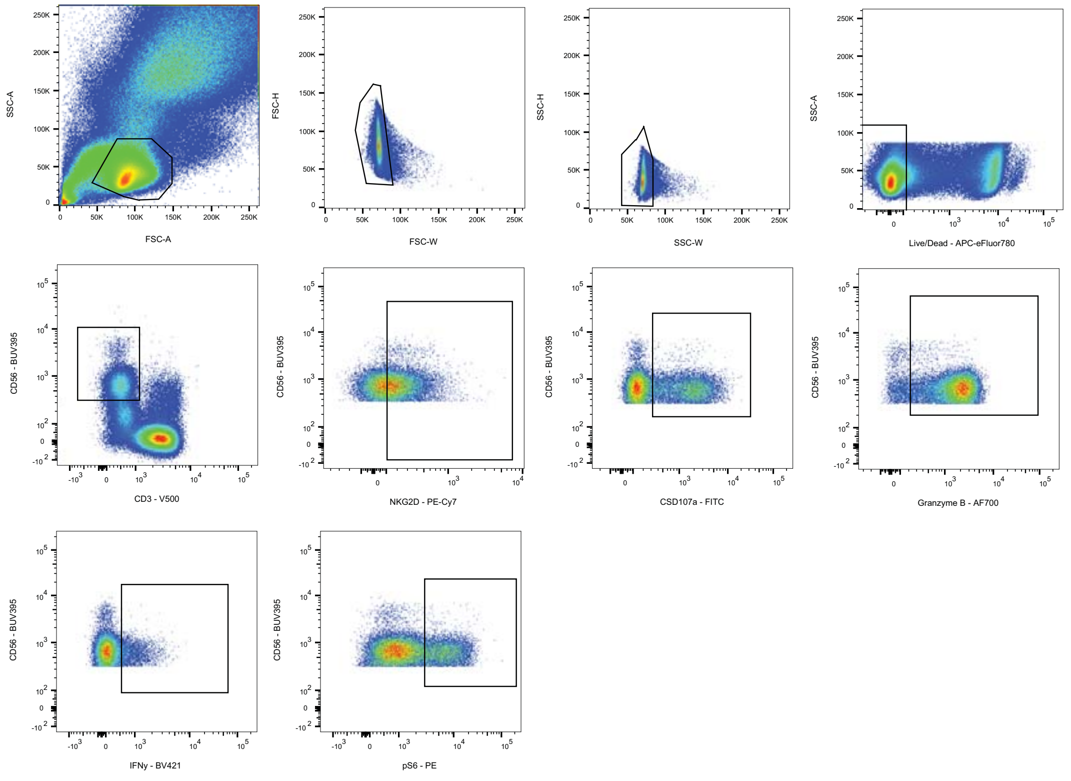

Supplemental Figure 5: Gating strategy for functional assays

Gating strategy for analysis of NK cell function of CLL patients and HC. (A) Example of cytotoxicity assay results by flow, from left to right and top to bottom: gating of target cells, gating of CellTrace Violet positive K562 cells, gating of live cells in target only control, or a co-culture with HC or CLL PBMC. (B) Example of staining and gating strategy for NK cell functional read-outs after co-culture with target cells, from left to right and top to bottom: gating of lymphocytes, single cells, live cells, CD56^{+}CD3^{-} NK cells, NKG2D, CD107a, granzyme B, IFNγ and pS6.
